# Integrated Analysis of the Transcriptome and Metabolome Reveals the Network Regulating Fruit Taste in Sponge Gourd (*Luffa cylindrica*)

**DOI:** 10.3390/foods14101753

**Published:** 2025-05-15

**Authors:** Yaqian Chai, Wenjing Qiu, Zhikun Li, Luyao Gao, Wenqi Dong, Peng Zhang, Shengjun Zhou, Xin Wang, Yuqiang Zhu, Yuyan Sun

**Affiliations:** 1 Institute of Vegetables, Zhejiang Academy of Agricultural Sciences, Hangzhou 310021, China; cyq15509465023@163.com (Y.C.); 17686260196@163.com (W.Q.); gaoluyao@zaas.ac.cn (L.G.); dwq9516@sina.com (W.D.); zhangp@zaas.ac.cn (P.Z.); zhousj@zaas.ac.cn (S.Z.); wangx8@zaas.ac.cn (X.W.); citarm@126.com (Y.Z.); 2 College of Agriculture, Shihezi University, Shihezi 832003, China; 3 Zhejiang Provincial Agricultural Technology Extension Center, Hangzhou 311300, China; wdlzk@sina.cn

**Keywords:** sponge gourd, taste, transcriptome, metabolome, mechanism

## Abstract

Sponge gourd fruit is highly favored by consumers because of its nutritional and medicinal properties. Continuous increases in living standards have led to an increase in the demand for high-quality fruits and vegetables. Hence, we explored the mechanisms that regulate fruit taste development. Specifically, two sponge gourd materials, ZS203 (GT) and ZAAS-106 (BT), which differ in fruit taste, were selected for transcriptomic and metabolomic analyses. Ascorbic acid, soluble solids, and crude protein contents were significantly higher in GT than in BT. Similarly, the lysine, phenylalanine, and tryptophan contents were higher in GT than in BT (1.48-, 1.60-, and 1.38 times higher, respectively). Transcriptomic analysis of GT and BT fruits identified 1821 upregulated and 1185 downregulated differentially expressed genes (DEGs) in GT, while metabolomic analysis detected 25 upregulated differentially accumulated metabolites (DAMs) and 28 downregulated DAMs in GT. A correlation analysis suggested that DAMs and DEGs related to vitamin B6 metabolism, tryptophan metabolism, and phenylalanine metabolism contribute to the differences in sponge gourd fruit taste; a potential mechanism underlying this diversity was proposed. Additionally, expression data for the 15 DEGs were consistent between transcriptomic and qRT-PCR analyses. Notably, this study revealed a potential mechanism for regulating differences in sponge gourd fruit taste, with possible implications for breeding novel varieties with optimized fruit taste.

## 1. Introduction

Sponge gourd, which belongs to the family Cucurbitaceae and genus *Luffa*, is an annual herbaceous climbing plant native to tropical and subtropical regions of Asia [1]. The genus *Luffa* includes nine species, among which *Luffa cylindrica* (L.) Roem. and *Luffa acutangula* (L.) Roxb. are cultivated species [2]. According to earlier research, sponge gourd is rich in amino acids, vitamins, dietary fiber, and trace elements, which help to explain its high nutritional value [3]. Sponge gourd also contains alkaloids, flavonoids, sterols, glycosides, and glycoproteins, which contribute to its high medicinal value [2,4]. These nutrients may also influence the sponge gourd fruit taste. However, the taste characteristics of sponge gourd fruit have not been as thoroughly characterized as those of fruits from other Cucurbitaceae crops (e.g., cucumber, melon, and watermelon) [5,6,7,8].

A desirable taste is a prerequisite for improving the quality of sponge gourd fruits. Taste is influenced by various substances, including organic acids, amino acids, sugars, vitamins, flavonoids, phenolic acids, and volatile compounds [9,10]. In a previous study on cucumber, 10 genes involved in the biosynthesis of sugars (fructose-6-phosphate and trehalose), linoleic acid, and amino acids (isoleucine, proline, and valine) were more highly expressed in grafted plants than in non-grafted plants [11]. In another study, an analysis of cucumber varieties that differ in terms of fruit flavors (i.e., better-tasting cucumber YX, North China-type cucumber KX, and pickled cucumber GX) identified some aldehydes and ketones as potentially important compounds for the superior taste of YX [5]. Watermelon fruit aroma is primarily influenced by volatile compounds [12]. Sweetness is a critical factor affecting the commercial appeal of watermelon fruit, with ongoing related research largely focusing on sucrose, fructose, and glucose [6]. The primary determinants of the sour taste of watermelon fruit are organic acids [7]. Melon grafting can significantly decrease the bitter amino acid concentrations in fruits [8]. Moreover, ethylene and aldehydes are key contributors to the aromatic profiles of melon fruits [13].

Increasing awareness of the health benefits of fruits has been accompanied by a growing demand for fruit and vegetable nutritional components, which have significant commercial value. Fruits with potent antioxidant activities are useful for mitigating chronic disease pathogenesis, especially cardiovascular disorders and malignancies [5]. Some nutrients, such as ascorbic acid, carotenoids, coumarins, and other phenolic compounds, are effective free radical scavengers [14,15]. A previous study on the nutritional content of melon (i.e., different improved varieties, local varieties, and wild types) from various regions in India showed that many local varieties have relatively high phenolic compound contents and free radical scavenging activities, with potentially significant implications for preventing cardiovascular diseases [15]. Watermelon fruit also contains antioxidants, including flavonoids, anthocyanins, and polyphenols, at levels high enough to promote human health [16]. The watermelon fruit rind is a rich source of carotenoids and phenolic compounds with antioxidant, anti-inflammatory, and cardiovascular disease-preventing properties, making it a promising source material for the development of functional foods [17].

The taste quality of horticultural products is influenced by agricultural management, genetics, and environmental conditions [10]. However, because of limited research, the regulatory network responsible for the diversity in fruit taste among sponge gourd materials remains largely unknown. In the present study, two sponge gourd materials, ZAAS-106 (BT) and ZS203 (GT), which differ significantly in terms of fruit taste quality, were analyzed. On the basis of integrated transcriptomic and metabolomic analyses, the key genes and metabolites contributing to sponge gourd fruit taste quality differences were determined. The results of this study may provide the theoretical basis for developing sponge gourd varieties with optimal fruit taste quality.

## 2. Materials and Methods

### 2.1. Plant Materials

Through the sensory tasting of 15 volunteers who were more sensitive to the sweetness, bitterness, and umami of sponge gourd, two materials, BT and GT, with significant differences in taste, were selected. Both materials were cultivated at the Yangdu Research and Innovation Base of Zhejiang Academy of Agricultural Sciences in China. Physicochemical analysis of the upper soil layer (0–20 cm) revealed the following properties: total nitrogen, 0.65 g kg^−1^; available nitrogen, 22.00 mg kg^−1^; total phosphorus, 0.84 g kg^−1^; available phosphorus, 54.65 mg kg^−1^; total potassium, 15.18 g kg^−1^; available potassium, 69.85 mg kg^−1^; organic matter, 15.35 g kg^−1^; and pH, 8.10. At 10 days post-pollination, sponge gourd fruits free of deformities and pests were selected and then peeled. The middle fruit section of each fruit was collected and quickly homogenized at room temperature before measuring the amino acid and soluble solid contents. Some of the collected fruit flesh was cut into small pieces, wrapped in aluminum foil, and stored at −80 °C for subsequent analyses of ascorbic acid and crude protein content; these samples were also used for transcriptomic and metabolomic analyses. Three biological replicates were prepared for transcriptomic and physiological analyses, and six biological replicates were prepared for metabolomic analysis.

### 2.2. Determination of Amino Acids Content

A 400 µL aliquot of the fruit homogenate was added to a 2 mL centrifuge tube, and 1000 µL of 4% sulfosalicylic acid solution was added to obtain a final volume of 1400 µL. The resulting solution was oscillated for 1 h on a shaker and then centrifuged at 15,000× *g* for 10 min. The supernatant (800 µL) was transferred to chromatography vials for the analysis of the free amino acid profile using a Hitachi L-8900 amino acid analyzer (Hitachi High-Technologies Corporation, Tokyo, Japan).

### 2.3. Determination of Soluble Solids, Ascorbic Acid, and Crude Protein Content

To determine the soluble solids content, the fruit homogenate was applied to the prism surface of a PAL-1 refractometer. For the analysis of ascorbic acid content, fruit flesh pieces that were stored at −80 °C were lyophilized and ground to a powder. Ascorbic acid content was determined using an AsA-1-W ascorbic acid assay kit (Suzhou Keming Biotechnology Co., Ltd., Suzhou, China). The crude protein content was measured as described previously [18].

### 2.4. Transcriptome Sequencing and Analysis

Total RNA was extracted using TRIzol reagent (Invitrogen, Carlsbad, CA, USA) and then analyzed using a NanoDrop 2000 spectrophotometer (Thermo Fisher Scientific, Waltham, MA, USA) to assess quality. A cDNA library was constructed using high-quality RNA [19]. After initial quantification using Qubit 2.0, the library was diluted to 1.5 ng L^−1^. The insert size of the library was determined using an Agilent 2100 Bioanalyzer (Agilent Technologies, Santa Clara, CA, USA). If the insert size was as expected, quantitative real-time polymerase chain reaction (qRT-PCR) analysis was conducted to determine the effective library concentration (>2 nM) to ensure that the library quality was sufficient for sequencing. Different libraries were pooled according to their effective concentrations after passing the quality checks. The samples were sequenced using an Illumina NovaSeq 6000 platform (SMART GENOMICS, Tianjin, China).

After sequencing, the data were screened for quality using the default parameters of fastq [20]. Low-quality reads (i.e., reads containing an adapter and/or more than five N bases and reads in which bases with Q ≤ 15 represented more than 40% of the entire sequence) were eliminated, and the retained clean reads were rapidly and accurately aligned to a sponge gourd reference genome (http://cucurbitgenomics.org/v2/organism/33, accessed on 29 January 2024) using HISAT2 software (version 2.2.1) [21]. The reference genome was used to determine the genomic positions of clean reads [1].

Reads mapped to genes were counted before calculating the number of reads and fragments per kilobase of transcript per million mapped reads (FPKM) values of genes using FeatureCounts [22]. Principal component analysis (PCA) was performed using BMKCloud (www.biocloud.net). DEGs were analyzed using DESeq2, with |log2 (fold-change)| > 2 and −log10 (*p*) < 0.05 set as the criteria for identifying significant DEGs [23]. Volcano plots were generated using the ggplot2 package in R (version 4.2.0). The ClusterProfiler package in R (version 4.2.0) was used to perform Gene Ontology (GO) and Kyoto Encyclopedia of Genes and Genomes (KEGG) enrichment analyses of the DEGs [24]. Bar charts presenting the results of the GO enrichment analysis were plotted using Python 3.6.6 (pandas 0.23.4), whereas bubble plots presenting the results of the KEGG enrichment analysis were plotted using the ggplot2 package in R (version 4.2.0). A cluster analysis of DEGs was performed, and the results were visualized using the ComplexHeatmap 2.12.0 package in R (version 4.2.0).

### 2.5. Metabolome Determination and Analysis

GT and BT sponge gourd fruit samples (100 mg) stored at −80 °C were ground to a powder and added to 1.5 mL centrifuge tubes. The ground material was resuspended in 500 µL of 80% methanol, after which the samples were mixed by vortexing and maintained in an ice bath for 5 min. The mixture was centrifuged at 15,000× *g* for 20 min at 4 °C. The supernatant was diluted to a methanol concentration of 53% and subjected to another round of centrifugation under identical conditions for 20 min. Finally, the supernatant was recovered for liquid chromatography-mass spectrometry (LC-MS) analysis [25].

For LC, chromatographic separation was performed using a Thermo Syncronis C18 column (2.1 mm × 100 mm, 1.7 µm particle size) (ThermoFisher, Waltham Massachusetts, USA) and a Dionex Ultimate 3000 Ultra-High-Performance Liquid Chromatography system (Thermo Fisher, Waltham, MA, USA). Mobile phase A contained water (with 0.1% formic acid (*v*/*v*) and 2 mmol L^−1^ ammonium formate), and mobile phase B consisted of acetonitrile. Gradient elution conditions were as follows: 0–1 min, 95% A; 1–5 min, 95–40% A; 5–8 min, 40–0% A; 8–11 min, 0% A; 11–14 min, 0–40% A; 11–15 min, 40–95% A; 15–18 min, 95% A.

For untargeted metabolomic analysis, a Thermo Q Exactive HF-X mass spectrometer (Thermo Fisher, Waltham, MA, USA) was used with an electrospray ionization (ESI) source and an ESI voltage of 2.8 kV in both positive and negative modes. The sheath and auxiliary gas flow rates were maintained at 35 and 10 arbs, respectively, and the capillary temperature was 320 °C. Full-scan spectra (*m*/*z* 70–1050) were acquired at a resolution of 70,000. Data-dependent MS/MS scans (*m*/*z* 100–1000) were triggered for the 10 most intense ions per cycle at a resolution of 17,500, with a stepped normalized collision energy (NCE) of 20, 40, and 60 V to increase the fragment ion coverage. Quality control samples were analyzed every six injections to ensure that the data were reproducible (<5% variation in retention times and peak intensities).

Raw data were processed using TraceFinder 3.2.0 (Thermo Fisher Scientific) with the following parameters: peak alignment: (1) retention time deviation tolerance: 0.2 min and (2) mass-to-charge ratio (*m*/*z*) tolerance: 5 ppm; metabolite identification: (1) MS1 level: precursor ions were matched against the SMART GENOMICS self-built database (Tianjin, China), with a mass error tolerance of 5 ppm and (2) MS2 level: fragment ions were compared with reference spectra in the mzCloud database (https://www.mzcloud.org), with a similarity score threshold of 70% (minimum of three matched fragments). The original quantitative data were standardized (TIC normalization and batch correction) to identify metabolites and determine their relative quantities.

Metabolites were annotated according to the KEGG database (https://www.genome.jp/kegg/pathway.html, accessed on 28 December 2023) and the HMDB database (https://hmdb.ca/metabolites, accessed on 28 December 2023). DAMs were identified according to the following criteria: VIP > 1 and |log2 (fold-change)| > 2. Partial least squares discriminant analysis (PLS-DA) was conducted using BMKCloud (VIP > 1.0 as the screening criterion), and the ggplot2 package in R (version 4.2.0) was used to generate volcano and bubble plots. The ComplexHeatmap package in R (version 4.2.0) was used for cluster analysis of DAMs and visualization of the results.

### 2.6. DEGs Validation via qRT-PCR

Total RNA was extracted using TransZol (TransGen Biotech, Beijing, China), as previously described [26], and cDNA was synthesized, as previously described [27]. Fifteen DEGs associated with sponge gourd fruit taste quality were selected for qRT-PCR analysis, which was performed using TB Green Premix EX Taq^TM^ (Takara, Kusatsu, Japan), as previously described [28]. Primer sequences are provided in Table S1.

### 2.7. Statistical Analysis

The data were processed using Microsoft Excel 2021 software. The independent student’s *t*-tests or analysis of variance (ANOVA) were performed using the agricolae package in R (version 4.2.0). Plots were created using Origin software (version 2024 b).

## 3. Results

### 3.1. Differences in Morphology and Compounds Between BT and GT

**Figure 1:**
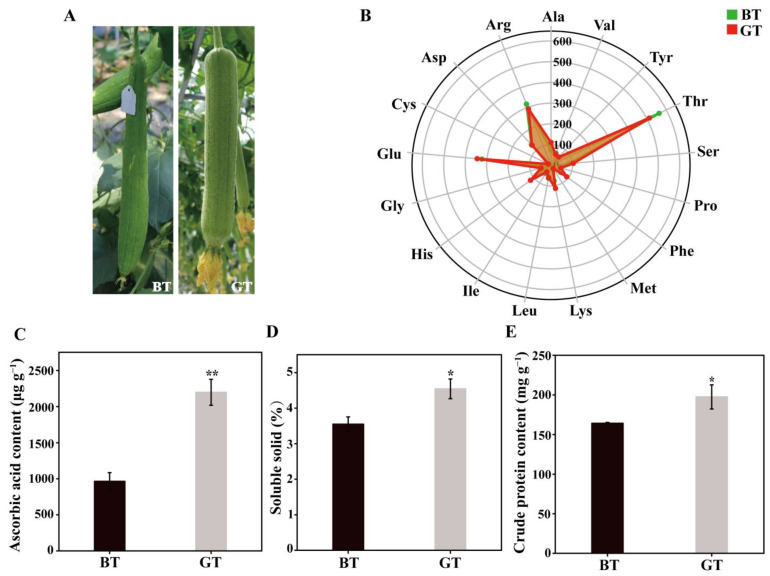
Differences Differences between BT and GT. (**A**) Phenotypic differences between BT and GT. (**B**) Differences in the amino acid content between BT and GT. (**C**) Differences in ascorbic acid content between BT and GT. (**D**) Differences in soluble solid content between BT and GT. (**E**) Differences in crude protein content between BT and GT. In (**C**–**E**), error bars represent the standard deviation of three replicates. Significant differences (* *p* < 0.05; ** *p* < 0.01) were calculated using Student’s *t*-test. The data in the column graphs are presented as the mean ± standard deviation (SD) of biological triplicates.

Our preliminary sensory and quality evaluations detected significant differences in fruit taste quality between BT and GT, which were cultivated under similar conditions (Figure 1A). The contents of ascorbic acid, soluble solids, crude protein, and various amino acids were measured (Figure 1B–E). The ascorbic acid, soluble solids, and crude protein contents in GT were 2198.61 μg g^−1^, 4.54%, and 197.56 mg g^−1^, which were 127.80%, 28.01%, and 20.19% higher than the corresponding contents in BT, respectively. Serine (Ser), glutamic acid (Glu), glycine (Gly), alanine (Ala), cysteine (Cys), tyrosine (Tyr), phenylalanine (Phe), lysine (Lys), histidine (His), and proline (Pro) contents in GT were 1.24-, 1.07-, 1.91-, 1.50-, 2.16-, 1.38-, 1.60-, 1.48-, 1.37-, and 1.16-times those of BT, respectively. By contrast, aspartic acid (Asp), threonine (Thr), valine (Val), methionine (Met), leucine (Leu), isoleucine (Ile), and arginine (Arg) contents were 1.03-, 1.10-, 1.09-, 1.14-, 1.07-, 1.22-, and 1.09-times higher in BT than in GT, respectively.

### 3.2. Analysis of Transcriptome Data

A total of 38.55 Gb of raw data were generated from the BT and GT libraries. After removing low-quality data, 37.47 Gb of clean data remained (97.20% of the total raw data). For clean data, Q30 exceeded 96.32%, with an average GC content of 44.94%. In addition, more than 97.28% of clean reads were mapped to genes. Accordingly, the transcriptome data were deemed appropriate for further analysis (Table S2).

**Figure 2:**
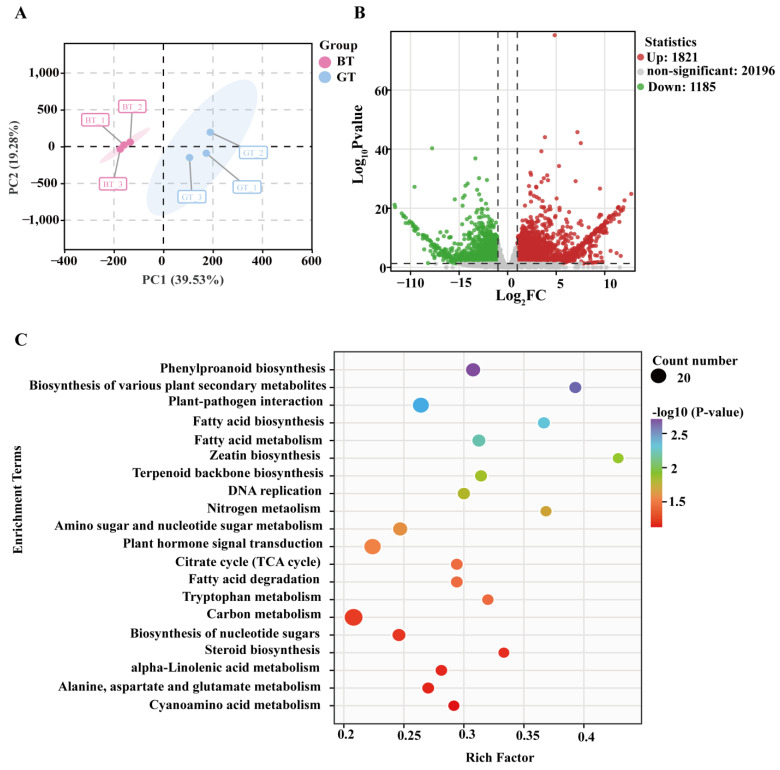
Analysis of transcriptome data. (**A**) PCA analysis. (**B**) Volcano plot of DEGs. (**C**) KEGG enrichment analysis for DEGs.

A PCA of the FPKM values indicated that the BT and GT samples were clearly separated along PC1 and PC2, which accounted for 39.53% and 9.28% of the total variance, respectively (Figure 2A). A total of 1821 upregulated and 1185 downregulated DEGs were identified in GT (relative to the corresponding expression levels in BT) (Figure 2B and Table S3). On the basis of a GO enrichment analysis, 371, 15, and 883 DEGs were annotated with terms from the biological process, cellular component, and molecular function categories, respectively (Figure S1). Moreover, the following KEGG pathways were significantly enriched among the 98 DEGs: phenylpropanoid biosynthesis (ath00940), fatty acid metabolism (ath00061), zeatin biosynthesis (ath00908), terpenoid backbone biosynthesis (ath00900), and tryptophan metabolism (ath00380) (Figure 2C). The expression levels of the 98 DEGs are shown in Figure S2.

### 3.3. Analysis of Metabolome Data

**Figure 3:**
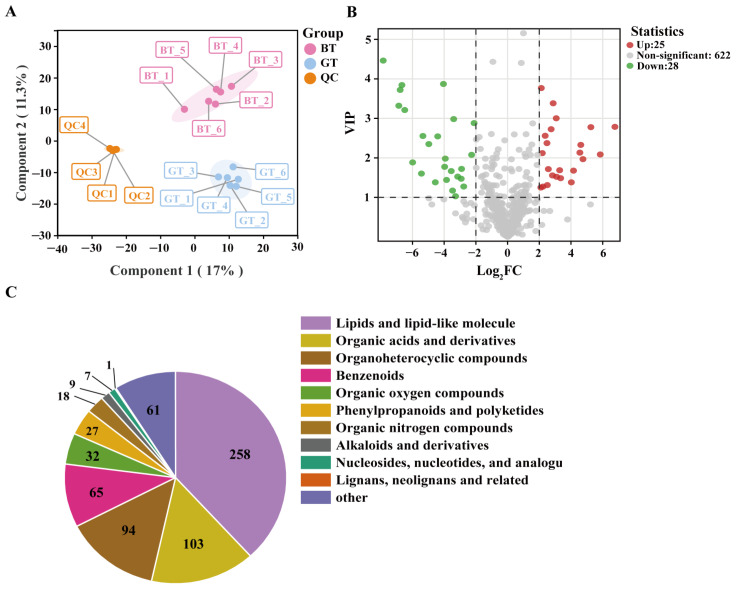
Analysis of metabolome data. (**A**) PCA. (**B**) Volcano plot of DAMs. (**C**) Classification of metabolites according to the HMDB database.

The PCA results indicated that component 1 and component 2 explained 16.78% and 12.22% of the total variance, respectively, demonstrating a clear separation between BT and GT (Figure 3A). A total of 675 metabolites were identified in BT and GT (Table S4), with lipids and lipid-like molecules being the most common metabolites (258), followed by organic acids and derivatives (103), organoheterocyclic compounds (94), benzenoids (65), organic oxygen compounds (32), phenylpropanoids and polyketides (27), organic nitrogen compounds (18), alkaloids and derivatives (9), nucleosides, nucleotides, and analogs (7), and Lignans, neolignans and related compounds (1). Additionally, 61 other metabolites were identified (Figure 3C).

Using VIP > 1 and |log2 (fold-change)| > 2 as criteria, 53 metabolites were identified as significant DAMs between BT and GT. A comparison with BT detected 25 upregulated DAMs in GT, such as pyridoxines (e.g., DL-lanthionine, N-oleoyl glycine, and pyridoxine) and tryptamines and derivatives (e.g., serotonin), as well as 28 downregulated DAMs in GT, including amino acids, peptides, and analogs (e.g., 6- (4-phenylpiperazino) hexanoic acid hydrochloride, pipecolic acid, and N-acetyl-aspartate), and pyridinecarboxylic acids and derivatives (Figure 3B and Table S5).

The annotated KEGG metabolic pathways among these DAMs included vitamin B6 metabolism (map00750), taste transduction (map04742), lysine degradation (map00310), phenylalanine metabolism (map00360), and tryptophan metabolism (map00380) (Table S6). More specifically, after removing duplicate metabolites, the following six DAMs were tentatively identified as being associated with these pathways: N-acetylmuramic acid (C304), pyridoxal (C98), serotonin (C87), hippuric acid (C455), normorphine (C278), and pipecolic acid (C112) (Figure S3).

### 3.4. Integrated Analysis of Transcriptome and Metabolome Results

**Figure 4:**
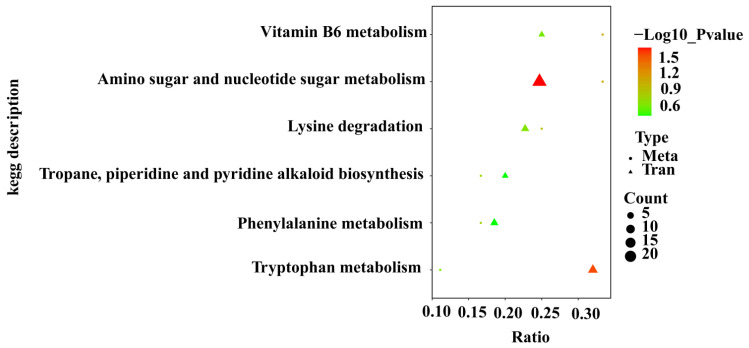
KEGG co-enrichment analysis of DEGs and DAMs.

A KEGG enrichment analysis of transcriptome and metabolome data indicated that the following pathways were annotated among 38 DEGs and five DAMs: vitamin B6 metabolism, lysine degradation, tropane, piperidine, and pyridine alkaloid biosynthesis, phenylalanine metabolism, and tryptophan metabolism (Figure 4).

**Figure 5:**
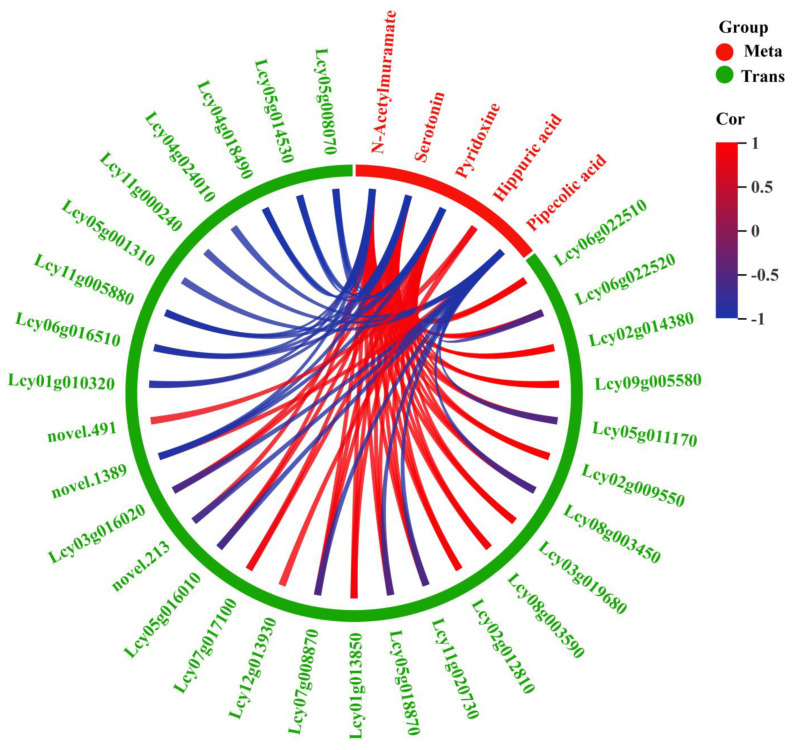
Correlation analysis of DAMs and DEGs. Note: Meta stands for DAMs and Trans stands for DEGs.

**Figure 6:**
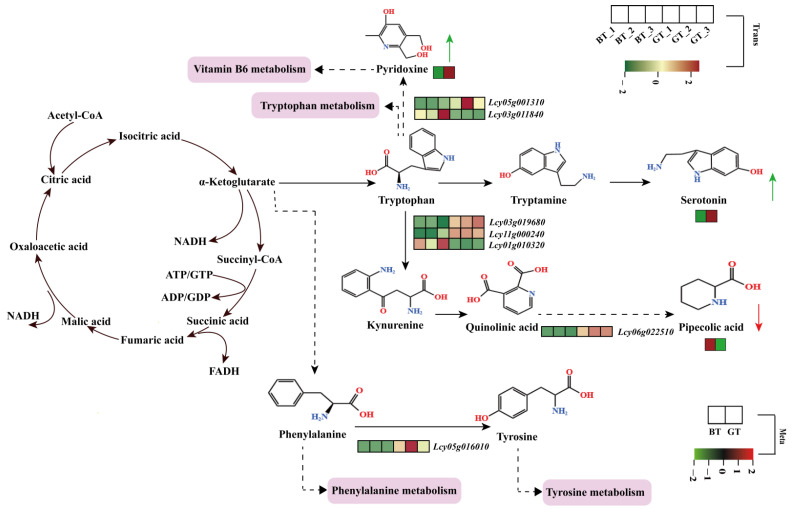
Potential mechanism model underlying the differences in fruit taste. In the figure, the solid straight arrows represent a single reaction, while the dotted straight arrows represent a multi-step reaction.

A correlation analysis of the above-mentioned 38 DEGs and five DAMs was performed, with *p* < 0.05 and Pearson correlation coefficient (R^2^) > 0.85. A total of 30 DEGs were significantly correlated with N-acetylmuramic acid, serotonin, vitamin B6, hippuric acid, and pipecolic acid (Figure 5). Among these DEGs, *Lcy05g016010*, *Lcy11g005880*, and *Lcy05g014530* were associated with phenylalanine metabolism; *Lcy08g003450*, *Lcy04g018490*, and *Lcy12g013930* were associated with lysine degradation; *Lcy06g022520* and *Lcy06g022510* were associated with tropane, piperidine and pyridine alkaloid biosynthesis; *Lcy03g019680*, *Lcy11g000240*, and *Lcy01g010320* were associated with tryptophan metabolism; and *Lcy11g020730* and *Lcy05g001310* were associated with vitamin B6 metabolism (Figure 6).

### 3.5. Potential Mechanism Model Underlying the Differences in Fruit Taste

Correlation analysis of DEGs and DAMs revealed that serotonin, pyridoxine, hippuric acid, and pipecolic acid are important determinants of sponge gourd fruit taste. An in-depth analysis of the pathways associated with these metabolites clarified the potential mechanisms underlying the differences in fruit taste between BT and GT (Figure 6). Notably, α-ketoglutarate serves as a substrate for a series of reactions that result in the synthesis of tryptophan, which is subsequently converted to serotonin via oxidation and decarboxylation. The upregulated expression of *Lcy03g019680*, *Lcy11g000240*, and *Lcy06g022510*, along with the downregulated expression of *Lcy01g010320* in GT, leads to the accumulation of pipecolic acid derived from tryptophan. Additionally, the upregulated expression of *Lcy05g001310* and the downregulated expression of *Lcy03g011840* in GT lead to an increase in the pyridoxine content. Intermediate α-ketoglutarate in the tricarboxylic acid cycle also generates phenylalanine through a series of complex reactions. In GT, phenylalanine is converted to tyrosine via the upregulated expression of *Lcy05g016010*.

### 3.6. Validation of DEGs via qRT-PCR

**Figure 7:**
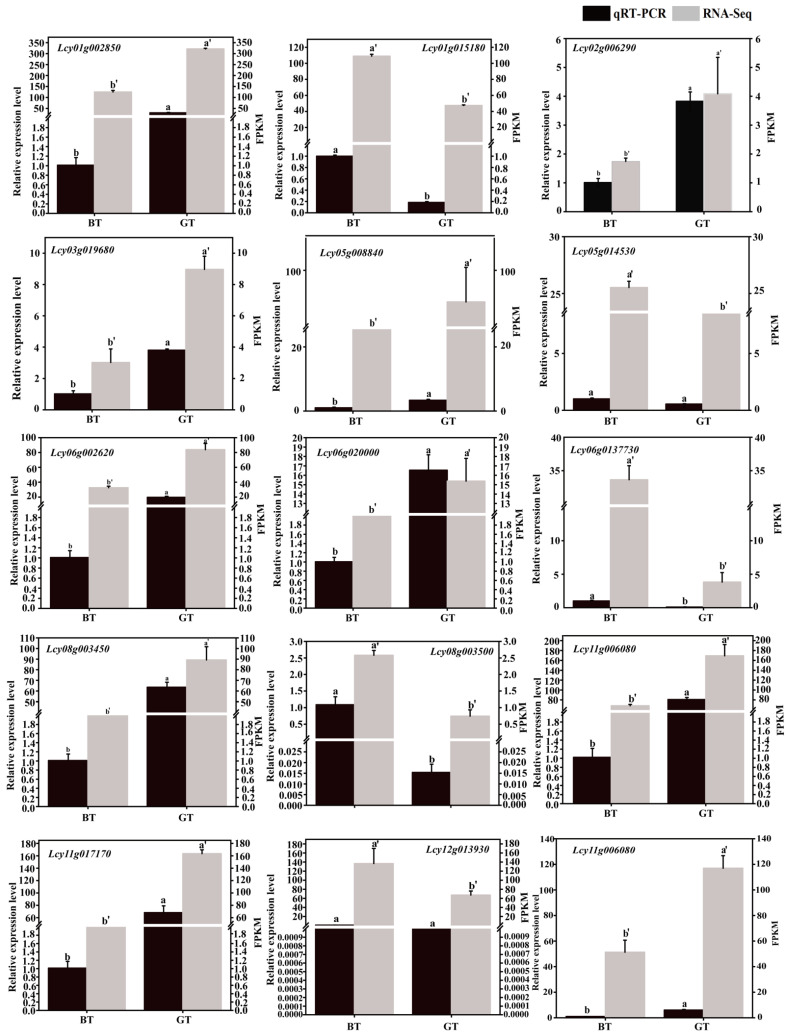
Expression levels of 15 DEGs as determined by qRT-PCR and transcriptome sequencing. In the figure, the error bar represents the standard deviation of three replicates. Different letters indicate significance as determined via ANOVA at *p* < 0.05; a and b indicate variations in qRT-PCR at GT and BT, whereas a′ and b′ indicate differences in RNA-Seq.

To assess the reliability of the transcriptome data, 15 DEGs were selected for qRT-PCR analysis (Figure 7). Specifically, 10 upregulated DEGs (*Lcy01g002850*, *Lcy02g006290*, *Lcy03g019680*, *Lcy05g008840*, *Lcy06g002620*, *Lcy06g020000*, *Lcy08g003450*, *Lcy11g006080*, *Lcy11g017170*, and *Lcy13g005740*) and five downregulated DEGs (*Lcy01g015180*, *Lcy05g014530*, *Lcy06g013730*, *Lcy08g003500*, and *Lcy12g013930*) in GT were included in the qRT-PCR analysis. The results showed that the qRT-PCR data for these 15 DEGs were consistent with the transcriptome sequencing data.

## 4. Discussion

Sponge gourd fruit is favored by consumers because of its high nutritional value and distinctive taste. The key nutritional components, such as amino acids, vitamins, phenolic acids, and flavonoids, vary among different materials [2,3,4], which helps explain the diversity in sponge gourd fruit taste [14,15]. To date, the DEGs and DAMs responsible for the differences in sponge gourd fruit taste have not been comprehensively analyzed. Our preliminary evaluation of sponge gourd materials (i.e., BT and GT) revealed significant differences in fruit taste and nutritional components, which compelled us to investigate the potential mechanism underlying the diversity in sponge gourd fruit taste. In this study, DEGs and DAMs associated with fruit taste differences between BT and GT were identified, and a potential mechanism involving three relevant pathways was proposed.

Pipecolic acid is a precursor of lysine [29]. The accumulation of pipecolic acid in fruits can enhance disease resistance, extend shelf life [30,31], and improve flavor [32]. A previous study involving metabolomic analysis and electronic tongue-based evaluation of taste detected a positive relationship between the acidity levels of three pomelo varieties and 12 amino acids (e.g., L-histidine and L-pipecolic acid) [33]. A study on dragon fruit showed that pipecolic acid is significantly more abundant in purple flesh than in white flesh, suggesting that pipecolic acid content may influence dragon fruit flavor [34]. In tomato, blocking the pipecolic acid biosynthetic pathway using genetic methods can delay fruit ripening and significantly decrease lycopene, sugar, and β-carotene content [32]. Furthermore, applying pipecolic acid to tomato reportedly leads to increases in sugar and β-carotene levels [32]. In the current study, the upregulated expression of *Lcy03g019680*, *Lcy11g000240*, and *Lcy06g022510* and the downregulated expression of *Lcy01g010320* in GT inhibited the accumulation of pipecolic acid. We also observed that the lysine content was 1.48 times higher in GT fruit than in BT fruit. These results suggest that more pipecolic acid may be converted to lysine in GT fruit than in BT fruit, thereby increasing the umami flavor of GT fruit [35]. Therefore, the enhanced umami taste of GT fruit may be related to a decrease in pipecolic acid levels in the fruit.

Pyridoxine is the most common form of vitamin B6 [36] and is an important nutrient in fruits and vegetables [37]. Additionally, the antioxidant activity of pyridoxine positively affects fruit freshness and taste [38]. For example, treating banana fruit with 9 mM pyridoxine can significantly increase the abundance of soluble sugars and carotenoids, which positively modulates fruit sweetness [39]. Cultivating tomato plants without soil can promote the absorption of nutrients and improve root aeration, which in turn stimulates the expression of genes associated with fruit flavor and increases the fruit’s vitamin B content [40]. Inoculating the soil with *Streptomyces pactum* Act12 during chili pepper plant cultivation can significantly increase the accumulation of vitamins and other flavor-related compounds, thereby optimizing pepper fruit taste [41]. In the present study, upregulated *Lcy05g001310* expression and downregulated *Lcy03g011840* expression promoted the accumulation of pyridoxine in GT fruit, which may help to explain why GT fruit has a superior taste and higher nutritional value than BT fruit.

Both phenylalanine and tyrosine are classified as aromatic amino acids on the basis of their flavor characteristics [42]. During the production of black tea, red-light withering can significantly increase phenylalanine and tyrosine content, thereby decreasing bitterness and astringency while enhancing aroma [43]. Phenolic compounds derived from phenylalanine positively influence tomato fruit flavor [44]. An earlier non-targeted metabolomic analysis of *Baccaurea ramiflora* Lour. showed that phenylalanine is more abundant in palatable red-fleshed fruit than in less palatable white-fleshed fruit [45]. In this study, the phenylalanine and tyrosine levels in GT determined by the automatic amino acid analyzer were 1.60- and 1.38 times those of BT, respectively, which may also be one of the reasons contributing to the superior taste quality of GT.

## 5. Conclusions

Transcriptomic and metabolomic analyses of GT and BT fruits identified 1821 upregulated and 1185 downregulated DEGs, as well as 25, upregulated and 28 downregulated DAMs in GT fruit. The results of the correlation analysis indicated that DAMs and DEGs related to vitamin B6 metabolism, tryptophan metabolism, and phenylalanine metabolism may help explain the diversity in sponge gourd fruit taste. On the basis of the study findings, a potential regulatory mechanism underlying the differences in fruit taste was proposed. The data presented herein may be applicable for breeding sponge gourd varieties with improved fruit taste.

## Data Availability

The original contributions presented in the study are included in the article/Supplementary Materials; further inquiries can be directed to the corresponding author.

## Supplementary Materials

The following supporting information can be downloaded at: https://www.mdpi.com/article/10.3390/foods14101753/s1, Table S1: Primer sequences for qRT-PCR of sponge gourd fruit. Table S2: Transcriptome sequencing results of GT and BT fruits. Table S3: Number of DEGs in GT vs BT. Table S4: The 675 metabolites identified in GT and BT sponge gourd fruit. Table S5: Identification of different metabolites in GT and BT of sponge gourd fruit. Table S6: Annotated KEGG metabolic pathways in GT vs BT. Figure S1: Bar plot of GO enrichment analysis for DEGs. Figure S2: Cluster analysis of 98 DEGs in BT and GT. Figure S3: Cluster analysis of six DAMs in BT and GT.
