# Supplementary figures and images for: Integrated Analysis of the Transcriptome and Metabolome Reveals the Network Regulating Fruit Taste in Sponge Gourd (Luffa cylindrica)
Source: Foods. 2025 May 15;14(10):1753. doi: 10.3390/foods14101753 (PMC12111081; doi:10.3390/foods14101753)

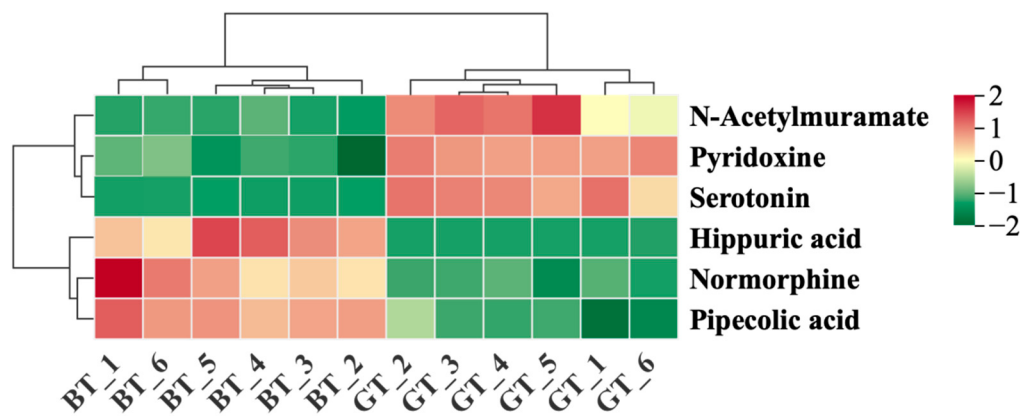

Figure S3 Cluster analysis of six DAMs in BT and GT.

Supplement: Supplementary file 1 [file foods-14-01753-s001.zip › Additional figure.pdf]
